# War Service for Resident Medical Officers

**Published:** 1915-08-28

**Authors:** E. R. Flint

**Affiliations:** Resident Surgical Officer. The General Infirmary, Leeds


### THE EDITOR'S LETTER-BOX.

### War Service for Resident Medical Officers.

*To the Editor of* The Hospital.

Sir,—I notice in The Hospital for August 7 a reference to The Hospital of July 17 with respect to commissions to be given to resident medical officers. Am I right in assuming from what was said at the meeting reported in The Hospital of August 7, that all resident medical officers at hospitals with teaching schools attached are to be given commissions at an early date? If this is so, can you tell me whether they will be Territorial or the ordinary R.A.M.C. commissions?

I shall be obliged to you if you can supply me with this information.—Yours faithfully,

E. R. Flint, Resident Surgical Officer.

The General Infirmary, Leeds, August 22.

[The scheme our correspondent refers to relates to hospitals with medical schools attached. Each school makes its own separate arrangement by communicating with the Director-General of Medical Services at the War Office. The Director-General of Medical Services has formulated a scheme whereby the members of the junior staff may receive temporary honorary commissions. On nomination to the junior staff the men wishing to hold commissions shall apply to the Dean of the Medical School, who issues a certificate of qualification to each officer to take to the War Office. If accepted by the War Office the men will be given honorary commissions and an allowance for their outfit. They are next seconded for a period of three months in order to act as residents at the hospital, during which time they are liable to be called up for active service at forty-eight hours' notice. Every man is required to sign a contract that he will serve for one year at home or abroad from the time he finishes his resident appointment. Our correspondent's course is to apply to the Dean of the Leeds Medical School, and, if necessary, to move the authorities of the Leeds General Infirmary to approach the War Office so as to secure the adoption of a scheme similar to those in force in connection with some of the London hospitals having clinical schools attached.—Ed. The Hospital.]

